# Triazol-substituted titanocenes by strain-driven 1,3-dipolar cycloadditions

**DOI:** 10.3762/bjoc.10.169

**Published:** 2014-07-17

**Authors:** Andreas Gansäuer, Andreas Okkel, Lukas Schwach, Laura Wagner, Anja Selig, Aram Prokop

**Affiliations:** 1 Kekulé-Institut für Organische Chemie und Biochemie der Rheinischen Friedrich-Wilhelms-Universität Bonn, Gerhard-Domagk-Straße 1, D-53121 Bonn, Germany; 2 Medizinische Klinik für Hämatologie, Onkologie und Tumorimmunologie Campus Vichow Klinikum Charité Berlin, Augustenburger Platz 1, D-13353 Berlin, Germany; 3 Abteilung für Kinderonkologie /-hämatologie Kinderkrankenhaus der Stadt Köln Amsterdamerstrasse 59, D-50735 Köln, Germany

**Keywords:** azides, click-chemistry, cycloadditions, cytotoxicity, titanocenes

## Abstract

An operationally simple, convenient, and mild strategy for the synthesis of triazole-substituted titanocenes via strain-driven 1,3-dipolar cycloadditions between azide-functionalized titanocenes and cyclooctyne has been developed. It features the first synthesis of titanocenes containing azide groups. These compounds constitute ‘second-generation’ functionalized titanocene building blocks for further synthetic elaboration. Our synthesis is modular and large numbers of the complexes can in principle be prepared in short periods of time. Some of the triazole-substituted titanocenes display high cyctotoxic activity against BJAB cells. Comparison of the most active complexes allows the identification of structural features essential for biological activity.

## Introduction

Group 4 metallocenes and derivatives of Cp_2_TiCl_2_, in particular, continue to be in the focus of contemporary research as a promising class of cytotoxic compounds [1–10], as efficient reagents and catalysts [11–16], as organometallic gelators [17–20], and in their own right [21–22]. In order to further investigate and improve these properties it is mandatory to access as yet unexplored functional titanocenes. This is most easily achieved with modular, efficient, and general strategies for the synthesis of these complexes. Classical approaches with metalation at the end of the sequence usually do not meet these requirements. This is because introduction of functional groups is difficult due to the nucleophilicity of the cyclopentadienyl anions before metalation and the electrophilicity of titanium after metalation [21–22].

**Scheme 1:**
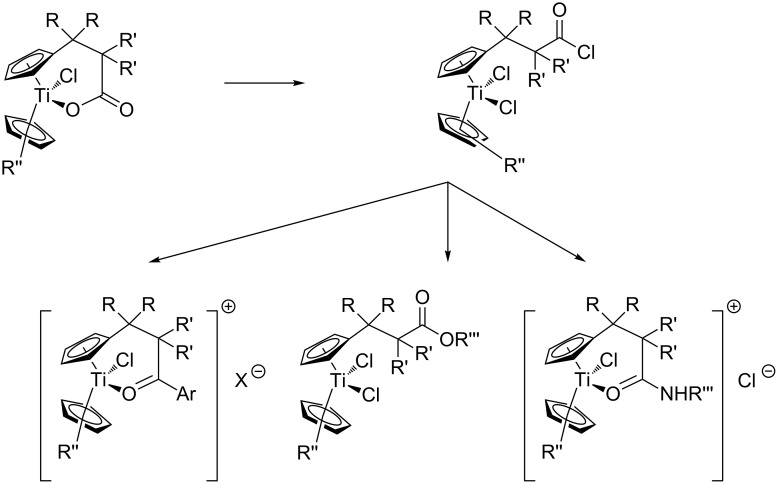
Modular titanocene synthesis via acylation reactions [24].

We have devised a conceptually different approach addressing these issues. It relies on the use of carboxylate-containing titanocene building blocks [23–25]. From these compounds the corresponding acid chlorides can be prepared by addition of SOCl_2_. The acid chloride group is more electrophilic than the [TiCl_2_] fragment and therefore many titanocenes containing ligands with pending amide, ester, and ketone groups can be prepared with classical organic acylation reactions (Friedel–Crafts reaction, esterification, amide synthesis, Scheme 1). Some of these complexes have been used as organometallic gelators [19–20], as a novel class of cytostatic compounds [26], and catalysts for unusual radical cyclizations [27–31]. The ketone and amide substituted catalysts are cationic due to the intramolecular coordination of the carbonyl group [30–31]. This feature is essential for the cytostatic and catalytic activity. Moreover, in the amide complexes hydrogen bonding of the N–H bond to chloride or [ZnCl_4_]^2−^ is a crucial structural feature [31]. The gelation ability of the neutral ester-substituted titanocenes critically depends on the steric demand of the substituents on the cyclopentadienyl ligands. The carboxylates are valuable complexes for mediating highly chemoselective Barbier type allylations [32–33].

These findings demonstrate that the properties of our functional titanocenes critically depend on both the direct environment of the Ti center and the periphery of the complex. Therefore, it is desirable to develop novel entries to titanocenes with even higher structural and functional diversity to improve the observed functions. An especially attractive approach is the use of already functionalized building blocks as starting materials in diversity oriented synthesis that increases the molecular complexity. Any synthetic methodology used in this context must take into account the sensitivity of the titanocene towards nucleophiles.

We decided to address these issues by employing cationic amide-substituted titanocenes as building blocks and strain-driven 1,3-dipolar cycloadditions [34–40] as synthetic methodology for the preparation of such ‘second-generation’ functional titanocenes. This line of action seemed especially appealing for two reasons. First, the cationic amide titanocenes have already displayed interesting activity and therefore serve as our lead structures. Second, the strain-driven 1,3-dipolar cycloadditions have evolved as extremely mild reactions for the functionalization of complex molecules. Since no metal complexes are required to catalyze the 1,3-dipolar cycloaddition [41–43], the reaction can be even used for the functionalization of biomolecules in living systems and has therefore been called bioorthogonal [34–35].

To the best of our knowledge, no examples of the Ti-containing substrates for our strategy, i.e., azide-functionalized titanocenes, have been reported in the literature. One aspect of our study is to establish if such complexes are stable and readily available in high yield. It should be noted that only a single example of a Cu-catalyzed 1,3-dipolar cycloaddition with an alkyne-functionalized titanocene has been described [44]. Therefore, the properties of triazol-substituted titanocenes, the products of the 1,3-dipolar cycloaddition, are also largely unexplored.

## Results and Discussion

### Synthesis of the titanocenes

#### Preparation of the starting materials

**Figure 1:**
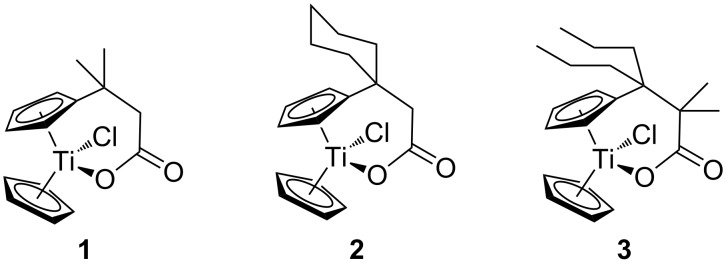
Carboxylates employed as titanocene starting materials for azide-substituted complexes.

We started our investigation with the preparation of azide-substituted cationic titanocenes. To this end, the titanocene carboxylates **1**–**3** shown in Figure 1 were employed as substrates because their substitution pattern should allow a first simple assessment of structure–activity relationships.

**Figure 2:**
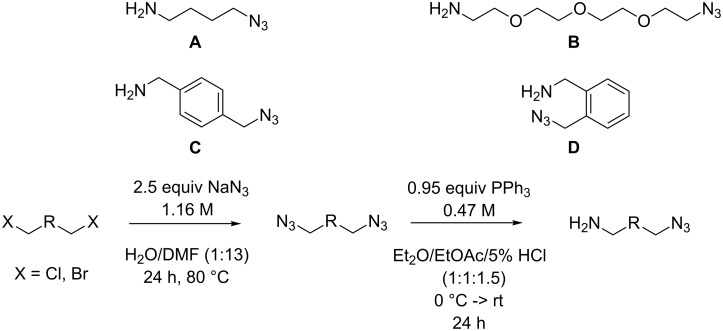
Azides employed in this study and conditions for their synthesis.

The compounds **A**–**D** shown in Figure 2 were used as amino-substituted azides. They are readily obtained from the corresponding diazides through a Staudinger reaction (see Supporting Information File 1 for details) [45–46]. As for the carboxylates the different tether lengths and substitution patterns of the arene allow to study the effect of substitution on the activity of the complexes. The ether tether in **B** serves as a model for PEG.

The titanocene carboxylates **1**–**3** were transformed into the corresponding acid chlorides and then reacted with amino azides **A**–**D** in the presence of NaH without purification of the acid chlorides. Typical results are summarized in Table 1.

**Table 1:** Synthesis of cationic titanocenes containing azides (yield over two steps, see Supporting Information File 1 for details).

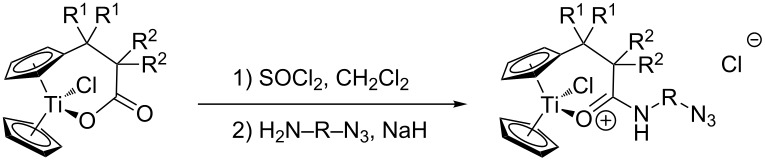		

substrates	product	yield/[%]

**1**, **A**	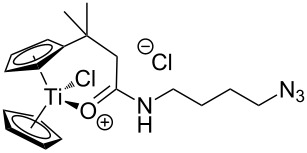 **4**	78
**1**, **B**	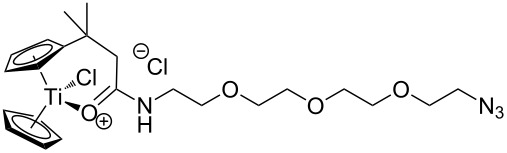 **5**	51
**1**, **C**	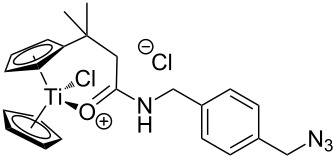 **6**	89
**2**, **A**	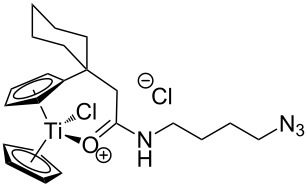 **7**	89
**2**, **C**	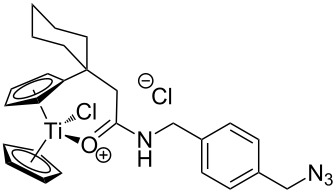 **8**	63
**2**, **D**	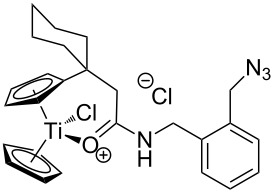 **9**	71
**3**, **B**	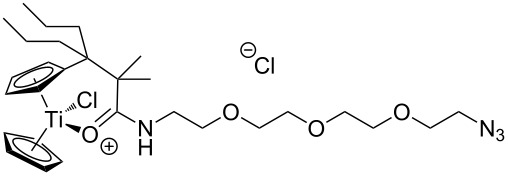 **10**	51
**3**, **D**	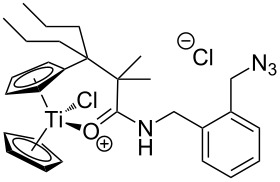 **11**	31

Gratifyingly, the acylation reactions proceed without problems and the azide-functionalized titanocenes can generally be obtained in good yields after 16 h. The somewhat lower yields obtained with carboxylate **3** are probably due to an increased bulkiness of the ligand’s substituents. It should be noted that polyether groups can be readily incorporated into cationic titanocenes. This suggests that the cationic titanocenes can be readily immobilized by covalent binding to PEG.

In general, our results clearly demonstrate that the azide group is compatible with cationic titanocenes. Moreover, it is obvious that large libraries of such titanocenes can be accessed from our carboxylates in short periods of time.

#### Strain-driven 1,3-dipolar cycloadditions

With the new titanocene building blocks in hand we turned our attention to their further functionalization through the strain-driven 1,3-dipolar cycloaddition with cyclooctyne. The original conditions of Wittig [36], the reaction of cyclooctyne with phenyl azide, and the numerous applications pioneered by Bertozzi suggest that the reaction proceeds under mild conditions [34–35,37–40]. Therefore, we simply mixed the titanocenes and cyclooctyne in CH_2_Cl_2_ at room temperature. The concentration of the substrates was intentionally kept low (0.1 M) to avoid a too intense evolution of heat. Typical examples of the reaction are summarized in Table 2.

**Table 2:** Strain-driven 1,3-dipolar cycloadditions between cyclooctyne and azide-functionalized titanocenes in CH_2_Cl_2_ (0.1 M).

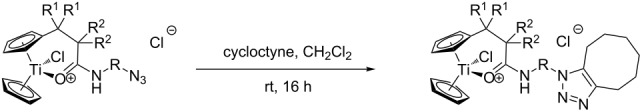		

titanocene	product	yield/[%]

**4**	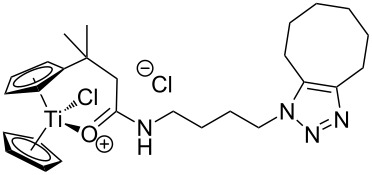 **12**	80
**5**	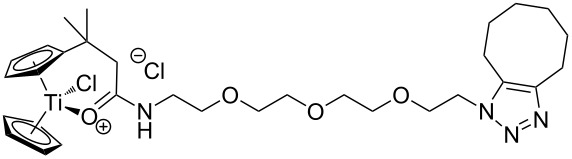 **13**	88
**6**	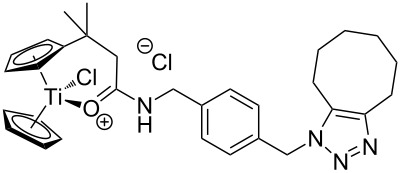 **14**	78
**8**	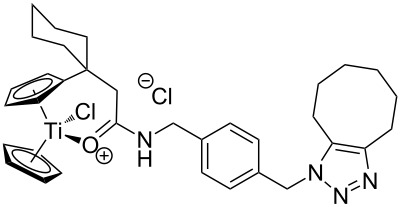 **15**	79
**9**	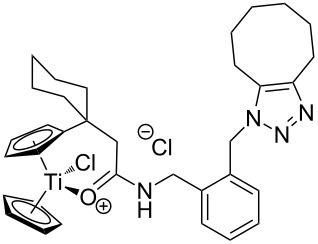 **16**	79
**10**	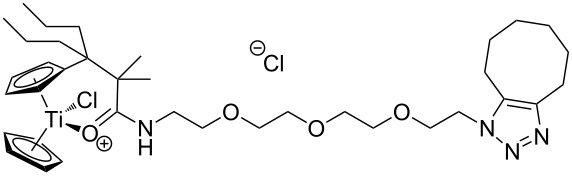 **17**	92
**11**	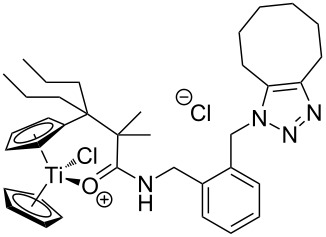 **18**	75

The results demonstrate that the strain-driven 1,3-dipolar cycloaddition is a convenient and very mild route to triazole-functionalized cationic titanocenes. The yields between 75% and 92% are satisfying. It should be noted that the polyether-substituted complexes **13** and **17** are obtained in high yield. This opens further interesting perspectives for the immobilization of titanocene complexes.

#### Cytotoxicity studies

One of the pertinent features of titanocenes is their cytotoxicity [1–5]. Therefore, we investigated this particular property of our novel complexes. To guarantee comparability with a previous study [26] we discuss our results of the lymphoma cell line BJAB. Cell surface transmembrane receptor CD95, through which apoptosis can be induced, is expressed by BJAB cells. Cell death can be induced in these cells both by the extrinsic and the intrinsic apoptosis-signalling pathway [47–49]. Therefore, BJAB cells are well-suited for studying the induction of apoptosis by our cationic titanocenes [50–53]. It is logical to study apoptosis induction, expressed as AC_50_ values, instead of nonspecific cytotoxicity, which is usually reported as LC_50_ values, because cytotoxic drugs operate by specific induction of apoptosis. So we determined the AC_50_ values of our titanocenes, i.e., the concentrations causing specific apoptosis in 50% of lymphoma cells, counting all cells with membrane damage.

**Figure 3:**
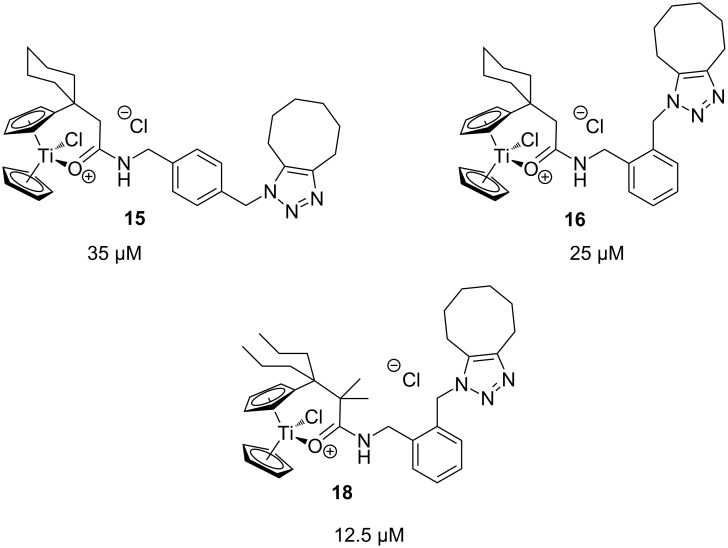
Most active titanocenes of this study and their AC_50_ values.

The azide-substituted complexes showed no significant apoptosis induction (AC_50_ > 100 µM). Introduction of the triazole ring through 1,3-dipolar cycloaddition markedly changes the activity of our titanocenes as a function of the substitution pattern. The most active complexes are highlighted in Figure 3.

Gratifyingly, **18**, together with a ketone-substituted titanocene, displays the highest activity against the BJAB cell line of our cationic carbonyl-substituted titanocenes. Comparison of the three most active complexes also allows the identification of structural features essential for cytotoxic activity. First, a bulky substitution of the cyclopentadienyl ligand is favorable. Second, positioning of the triazol in close proximity – ortho-substitution in **16** leads to a lower AC_50_ value than para-substitution in **15** – of the metal center enhances the biological activity.

## Conclusion

In summary, we have devised an operationally simple, convenient, and mild strategy for the synthesis of triazole substituted titanocenes via strain-driven 1,3-dipolar cycloadditions between azide-functionalized titanocenes and cyclooctyne. It features the first synthesis of titanocenes containing azide groups. These compounds constitute functionalized ‘second-generation’ titanocene building blocks for further synthetic elaboration. Our synthesis is modular and large numbers of the complexes can in principle be prepared in short periods of time. Some of the triazole-substituted titanocenes display high cyctotoxic activity against BJAB cells. Comparison of the most active complexes allows the identification of structural features essential for biological activity.

Future studies will concentrate on the preparation of neutral and of enantiomerically pure triazole-substituted titanocenes, also via metal catalyzed cycloadditions, for further increasing the biological activity of the complexes and for applications in enantioselective [54–55] cyclizations [56–58].

## Supporting Information

File 1 Experimental procedures and compound characterization, cytotoxicity studies.
