# Supplementary material for: Triazol-substituted titanocenes by strain-driven 1,3-dipolar cycloadditions
Source: Beilstein J Org Chem. 2014 Jul 17;10:1630–7. doi: 10.3762/bjoc.10.169 (PMC4142980; doi:10.3762/bjoc.10.169)
Supplement: File 1 — Experimental procedures and compound characterization, cytotoxicity studies. [file Beilstein_J_Org_Chem-10-1630-s001.pdf]

Supporting Information

for

Triazol-substituted titanocenes by strain-driven 1,3-dipolar cycloadditions

Andreas Gansäuer*[1], Andreas Okkel[1], Lukas Schwach[1], Laura Wagner[2], Anja Selig[2], Aram Prokop[3]

Address: [1]Kekulé-Institut für Organische Chemie und Biochemie der Rheinischen Friedrich-Wilhelms-Universität Bonn, Gerhard-Domagk-Straße 1, D-53121 Bonn, [2]Medizinische Klinik für Hämatologie, Onkologie und Tumorimmunologie Campus Vichow Klinikum Charité Berlin, Augustenburger Platz 1, D-13353 Berlin and [3]Abteilung für Kinderonkologie /-hämatologie Kinderkrankenhaus der Stadt Köln Amsterdamerstrasse 59, D-50735 Köln

Email: Andreas Gansäuer - andreas.gansaeuer@uni-bonn.de

Experimental procedures and compound characterization, cytotoxicity studies

Experimental Section:

All starting materials were purchased from commercial sources and used as received unless stated otherwise. Dichloromethane was dried prior to use over CaH_2. Diazides and amino azides **A**, **B**, **C**, **D** were synthesised as reported in literature [1-4].

Physical measurements and instrumentation:

^{1}H NMR and ^{13}C NMR spectra were recorded on a DPX 300 and DPX 400 Bruker spectrometer; the chemical shifts (in ppm) are reported relative to nondeuterated solvent residual as reference. EI mass spectra were recorded on a MS 50 spectrometer from Kratos as well as on a MAT 95 spectrometer from Thermoquest. ESI mass spectra were recorded on a micrOTOF-Q spectrometer from Bruker Daltonik and IR spectra were recorded on an ATR Nicolet 380 spectrometer from Thermo Electron. Melting Points were measured on a Büchi 530 Melting Point and are uncorrected.

Determination of cell concentration and cell viability:

In a similar manner as described in [5], cell viability was determined by CASY® Cell Counter + Analyzer System of Schaerfe System GmbH (Reutlingen, Germany). Settings were specifically defined for the requirements of the used cells. With this system the cell concentration is analyzed simultaneously in three different size ranges: cell debris, dead cells, and viable cells were determined in one measurement. BJAB cells were seeded at a density of 1 Å~ 105 cells/mL and treated with different concentrations of a titanocene-derivate, non treated cells served as controls. After 24 h of incubation at 37 °C, 5% CO_2, cells were resuspended properly and 100 µL of each well was diluted in 10 mL CASYton (ready-to-use isotonic saline solution) for an immediate automated count of the cells.

Measurement of DNA fragmentation:

In a similar manner as described in [6], apoptotic cell death was determined by a modified cell cycle analysis, which detects DNA fragmentation on the single cell level. For measurement of DNA fragmentation cells were seeded at a density of 1 Å~ 105 cells/mL and treated with different concentrations of a titanocene-derivate. After 72 h of incubation at 37 °C, 5% CO_2, cells were collected by centrifugation at 1500 rpm for 5 min, washed with PBS at 4 °C, and fixed in PBS/2% (v/v) formaldehyde on ice for 30 min. After fixation, cells were incubated with ethanol/PBS (2:1, v/v) for 15 min, pelleted, and resuspended in PBS containing 50 µg/mL RNase A. After incubation for 30 min at 37 °C, cells were pelleted again and finally resuspended in PBS containing 50 µg/mL propidium iodide. Nuclear DNA fragmentation was then quantified by flow cytometric determination of hypodiploid DNA. Data were collected and analyzed using a FACScan (Becton Dickinson, Heidelberg, Germany) equipped with the CELLQuest software. Data are given in percentage of hypoploidy (subG1), which reflects the number of apoptotic cells.

AnnexinV-propidium iodide binding assay:

In a similar manner as described in [7], early apoptotic rates were assessed with flow

cytometry using the annexin V–fluorescein isothiocyanate/propidium iodide (PI) kit (BD Pharmingen, San Diego, CA, USA), in which annexin Vbound to exposed phosphatidylserine of the early apoptotic cells, whereas PI stained the cells that had an increased membrane permeability, i.e., the late apoptotic cells. Samples were prepared according to the manufacturer's instructions. Flow cytometry analysis was performed using a FACS-Calibur cytometer (Becton Dickinson, Heidelberg, Germany). The annexin-V+/PI- cells were defined as early apoptotic cells.

General procedure for the synthesis of azide-functionalized titanocenes from carboxylates

To a solution of the carboxylate (1 equiv.) in CH_2Cl_2 (3 mL/mmol) was added $SOCl_2$ (3 mL/mmol). After stirring for 3 h at r.t. excess $SOCl_2$ and solvent was removed in vacuo for 6 h at 45 °C. The resulting acid chloride was dissolved in CH_2Cl_2 (6 mL/mmol) and transferred via syringe to a suspension of NaH (10 equiv.) and amino azide (2 equiv.) in CH_2Cl_2 (10 mL/mmol). Stirring was continued for 16 h at r.t.. After filtration through Celite the volatiles were removed under reduced pressure and the residue was chromatographed on BioBeads S-X3 to yield the desired product.

Synthesis of **4**: Carboxylate (313 mg, 1 mmol), $SOCl_2$ (3 mL), NaH (240 mg, 10 equiv.) and **A** (228 mg, 2 equiv.) to yield **4** (347 mg, 78%) over two steps;

M.p.: 72-76 °C (decomposition); ^{1}H-NMR (400 MHz, $CDCl_3$): δ = 11.93 (br. s, 1H), 7.21 – 7.13 (m, 1H), 7.00 – 6.86 (m, 1H), 6.68 (s, 5H), 6.60 – 6.53 (m, 1H), 5.95 – 5.89 (m, 1H), 3.30 (t, J = 6.0 Hz, 2H), 3.38 – 3.24 (m, 1H), 3.26 – 3.15 (m, 2H), 2.97 – 2.85 (m, 1H), 1.72 – 1.56 (m, 4H), 1.24 (s, 6H);^{13}C-NMR (100 MHz, $CDCl_3$): δ = 176.5, 150.2, 125.4, 121.4, 119.5, 117.2, 109.3, 51.0, 46.3, 41.2, 34.6, 30.3, 26.4, 26.4, 26.0; MS: (10.0 eV, ESI): m/z (%) = 405.2 (100); HRMS (10.0 eV, ESI): calcd. for $C_{20}H_{29}N_4O_2{}^{48}Ti^+$: 405.1766; found: 405.1765 [M – 2Cl + OCH_3]$^+$); IR: ATR, v [cm^{-1}] = 2935, 2870, 2090, 1610, 1550, 1440, 1370, 1285, 1190, 1000, 825, 730, 415. Crystallized from CH_2Cl_2, Anal. calcd. for $C_{19}H_{26}Cl_2N_4OTi$ (CH_2Cl_2): C 49.89, H 5.76, N 12.12; found: C 49.69, H 6.02, N 12.04.

Synthesis of **5:** Carboxylate (313 mg, 1 mmol), $SOCl_2$ (3 mL), NaH (240 mg, 10 equiv.) and **B** (436 mg, 2 equiv.) to yield **5** (280 mg, 51%) over two steps;

M.p.: 155 °C (decomposition); ^{1}H-NMR (400 MHz; $CDCl_3$): δ = 11.92 (br. s, 1H), 7.04 (s, 1H), 6.83 - 6.79 (m, 1H), 6.70 (s, 5H), 6.58 (s, 1H), 5.98 (m, 1H), 3.74 - 3.56 (m, 13H), 3.49-3.39 (m, 1H), 3.38 (t, J = 5.1 Hz, 3H), 3.22 (d, J = 14.3 Hz, 1H), 2.91 (d, J = 14.4 Hz, 1H), 1.28 (s, 6H); ^{13}C-NMR (100 MHz; $CDCl_3$): 176.3, 150.2, 124.9, 121.3, 120.0, 116.4, 108.9, 70.7, 70.6, 70.5, 70.4, 70.08, 68.6, 50.8, 46.5, 41.5, 34.4, 29.6, 26.6; MS (2.0 eV, ESI): m/z (%) = 509.2 (100), 475.2 (8); HRMS (2.0 eV, ESI): cacld. for $C_{20}H_{29}N_4O_3{}^{46}Ti^+$: 543.1854; found 543.1851 [M – Cl + OCH_3]$^+$; IR: ATR, v [cm^{-1}] = 2922, 2871, 2023, 1615, 1556, 1102, 826, 417; Crystallized from CH_2Cl_2, Anal. calcd. for $C_{23}H_{34}Cl_2N_4OTi$ (CH_2Cl_2): C 45.45, H 5.72, N 8.83; found: C 45.52, H 5.82, N 8.84.

Synthesis of **6:** Carboxylate (626 mg, 2 mmol), $SOCl_2$ (6 mL), NaH (480 mg, 10 equiv.) and **C** (649 mg, 2 equiv.) to yield **6** (878 mg, 89%) over two steps;

M.p.: 185 °C (decomposition); ^{1}H-NMR (400 MHz, $CDCl_3$): δ = 12.78 (br. s, 1H), 7.44 (d, J = 8.1 Hz, 2H), 7.30 (d, J = 8.1 Hz, 2H), 6.94 – 6.90 (m, 1H), 6.86 - 6.82 (m, 1H), 6.54 – 6.49 (m, 1H), 6.46 (s, 5H), 6.03 – 5.99 (m, 1H), 4.51 (dd, J = 14.5 Hz, J = 6.4 Hz, 1H), 4.36 (dd, J = 14.5 Hz, J = 5.6 Hz, 1H), 4.30 (s, 2H), 3.33 (d, J = 14.4 Hz, 1H), 2.94 (d, J = 14.4 Hz, 1H), 1.28 (s, 3H), 1.20 (s, 3H); ^{13}C-NMR (100 MHz, $CDCl_3$): δ = 176.6, 150.7, 136.8, 135.1, 128.9, 128.6, 124.7, 121.2, 120.7, 116.2, 109.4, 54.4, 47.1, 44.9, 34.8, 30.1, 26.1; MS (10.0 eV, ESI): m/z (%) = 453.2 (100); HRMS (10.0 eV, ESI): calcd. for $C_{24}H_{29}N_4O_2{}^{48}Ti^+$: 453.1767; found: 453.1768 [M – 2Cl + OCH_3]$^+$); IR: ATR, v [cm^{-1}] = 2920, 2095, 1605, 1550, 1445, 1370, 1235, 1205, 1025, 825, 680, 560. Crystallized from CH_2Cl_2, Anal. calcd. for $C_{23}H_{26}Cl_2N_4OTi$ (CH_2Cl_2): C 54.61, H 5.22, N 10.98; found: C 54.61, H 5.30, N 10.99.

Synthesis of **7**: Carboxylate (352 mg, 1 mmol), $SOCl_2$ (3 mL), NaH (240 mg, 10 equiv.) and **A** (228 mg, 2 equiv.) to yield **7** (436 mg, 89%) over two steps;

M.p.: 108 °C; ^{1}H-NMR (400 MHz, $CDCl_3$): δ = 12.30 (br. s, 1H), 6.99 – 6.94 (m, 1H), 6.94 – 6.90 (m, 1H), 6.66 (s, 5H), 6.63 – 6.58 (m, 1H), 6.13 – 6.08 (m, 1H), 3.45 (d, J = 13.8 Hz, 1H), 3.34 (t, J = 6.3 Hz, 2H), 3.38 – 3.29 (m, 1H), 3.28 – 3.19 (m, 1H), 2.78 (d, J = 13.9 Hz, 1H), 1.84 – 1.53 (m, 10H), 1.50 – 1.31 (m, 3H), 1.24 – 1.11 (m, 1H); ^{13}C-NMR (75 MHz, $CDCl_3$): δ = 176.4, 150.7, 125.4, 121.2, 121.1, 116.0, 110.2, 51.0, 46.1, 41.0, 38.8, 38.4, 34.1, 26.4, 26.0, 25.4, 22.2, 21.7; MS (10.0 eV, ESI): m/z (%) = 445.2 (100); HRMS (8.0 eV, ESI): calcd. for $C_{23}H_{33}N_4O_2{}^{48}Ti^+$: 445.2080; found: 445.2078 [M – 2Cl + OCH_3]$^+$); IR: ATR, v [cm^{-1}] = 2925, 2850, 2090, 1610, 1550, 1440, 1375, 1000, 825, 730. Crystallized from CH_2Cl_2, Anal. calcd. for $C_{22}H_{30}Cl_2N_4OTi$ (CH_2Cl_2): C 51.81, H 5.98, N 10.79; found: C 52.04, H 6.27, N 10.92.

Synthesis of **8**: Carboxylate (352 mg, 1 mmol), $SOCl_2$ (3 mL), NaH (240 mg, 10 equiv.) and **C** (324 mg, 2 equiv.) to yield **8** (334 mg, 63%) over two steps;

M.p.: 108 °C; ^{1}H-NMR (400 MHz, $CDCl_3$): δ = 12.76 (t, J = 5.5 Hz, 1H), 7.44 (d, J = 8.0 Hz, 2H), 7.29 (d, J = 8.0 Hz, 2H), 7.11 – 7.05 (m, 1H), 6.92 – 6.87 (m, 1H), 6.49 (s, 5H), 6.45 – 6.41 (m, 1H), 6.17 – 6.11 (m, 1H), 4.47 (dd, J = 14.2 Hz, J = 6.2 Hz, 1H), 4.32 (dd, J = 13.9 Hz, J = 5.4 Hz, 1H), 4.29 (s, 2H), 3.46 (d, J = 13.3 Hz, 1H), 2.76 (d, J = 13.4 Hz, 1H), 1.86 – 1.05 (m, 10H); ^{13}C-NMR (100 MHz, $CDCl_3$): δ = 176.6, 150.6, 137.0, 135.1, 128.8, 128.8, 124.8, 122.4, 121.1, 115.2, 110.6, 54.5, 46.9, 44.9, 38.7, 38.6, 33.9, 25.3, 22.3, 21.7; MS (8.0 eV, ESI): m/z (%) = 493.2 (100), 527.2 (5); HRMS (8.0 eV, ESI): m/z: calcd. for $C_{27}H_{33}N_4O_2{}^{46}Ti^+$: 491.2124; found: 491.2124 [M – 2Cl + OCH_3]$^+$); IR: ATR, v [cm^{-1}] = 2925, 2850, 2090, 1605, 1555, 1435, 1375, 1240, 1020, 825, 730, 685. Crystallized from CH_2Cl_2, Anal. calcd. for $C_{26}H_{30}Cl_2N_4OTi$ (CH_2Cl_2): C 57.18, H 5.57, N 10.18; found: C 57.13, H 5.57, N 10.24.

Synthesis of **9**: Carboxylate (530 mg, 1.5 mmol), $SOCl_2$ (4.5 mL), NaH (360 mg, 10 equiv.) and **D** (486 mg, 2 equiv.) to yield **9** (635 mg, 71%) over two steps;

M.p.: 108 °C (decomposition); ^{1}H-NMR: (400 MHz, $CDCl_3$): δ = 12.76 (t, J = 5.5 Hz, 1H), 7.44 (d, J = 8.0 Hz, 2H), 7.29 (d, J = 8.0 Hz, 2H), 7.11 – 7.05 (m, 1H), 6.92 – 6.87 (m, 1H), 6.49 (s, 5H), 6.45 – 6.41 (m, 1H), 6.17 – 6.11 (m, 1H), 4.47 (dd, J = 14.2 Hz, J = 6.2 Hz, 1H), 4.32 (dd, J = 13.9 Hz, J = 5.4 Hz, 1H), 4.29 (s, 2H), 3.46 (d, J = 13.3 Hz, 1H), 2.76 (d, J = 13.4 Hz, 1H), 1.86 – 1.05 (m, 10H); ^{13}C-NMR (100 MHz, $CDCl_3$): δ = 176.6, 150.6, 137.0, 135.1, 128.8, 128.8, 124.8, 122.4, 121.1, 115.2, 110.6, 54.5, 46.9, 44.9, 38.7, 38.6, 33.9, 25.3, 22.3, 21.7; MS: (8.0 eV, ESI): m/z (%) = 493.2 (100), 527.2 (5); HRMS (8.0 eV, ESI): calcd. for $C_{27}H_{33}N_4O_2{}^{46}Ti^+$: 491.2124; found 491.2124 $[M – 2Cl + OCH_3]^+$; Crystallized from CH_2Cl_2, Anal. calcd. for $C_{26}H_{30}Cl_2N_4OTi$ (CH_2Cl_2): C 57.18, H 5.57, N 10.18; found: C 57.15, H 5.93, N 10.12.

Synthesis of **10**: Carboxylate (396 mg, 1 mmol), $SOCl_2$ (3 mL), NaH (240 mg, 10 equiv.) and **B** (436 mg, 2 equiv.) to yield **10** (323 mg, 51%) over two steps;

M.P. 165 °C (decomposition); ^{1}H-NMR (400 MHz; $CDCl_3$): δ = 11.07 (br. s, 1H), 7.14 (m, 1H), 6.70 (s, 5H), 6.49 - 6.42 (m, 1H), 6.33 (m, 1H), 5.98 (m, 1H), 3.91 - 3.83 (m, 1H), 3.74 - 3.56 (m, 12H), 3.50 - 3.42 (m, 1H), 3.37 (t, J = 4.5 Hz, 2H), 1.79 - 1.44 (m, 4H), 1.70 (s, 3H), 1.39 – 1.17 (m, 3H), 1.17 (s, 3H), 0.89 (t, J = 7.1 Hz, 3H), 0.82 (t, J = 7.1 Hz, 3H), 0.64 (m, 1H); ^{13}C-NMR (100 MHz; $CDCl_3$): δ = 183.1, 149.9, 126.5, 120.8, 119.9, 116.9, 109.3, 70.7, 70.6, 70.4, 70.3, 70.0, 68.9, 50.8, 47.7, 46.6, 42.3, 39.4, 39.1, 23.8, 21.9, 17.8, 17.5, 15.2, 15.03; MS (8.0 eV, ESI): m/z (%) = 593.3 (100), 579.3 (48). HRMS (8.0 eV, ESI): calcd. for $C_{30}H_{49}N_4O_5{}^{48}Ti^+$: 593.3182, found: 593.3180 $[M – 2Cl + OCH_3]^+$; IR: ATR, v [cm^{-1}] = 2958, 2869, 2098, 1585, 1525, 1440, 1348, 1282, 1022; Crystallized from CH_2Cl_2, Anal. calcd. for $C_{27}H_{46}Cl_2N_4O_4Ti$ (CH_2Cl_2): C 50.16, H 6.73, N 7.80; found: C 50.19, H 6.77, N 7.83.

Synthesis of **11**: Carboxylate (794 mg, 2 mmol), $SOCl_2$ (6 mL), NaH (480 mg, 10 equiv.) and **D** (649 mg, 2 equiv.) to yield **11** (357 mg, 31%) over two steps;

M.p.: 94 °C (decomposition); ^{1}H-NMR (400 MHz, $CDCl_3$): δ = 11.88 (br. s, 1H), 7.39 – 7.27 (m, 4H), 7.19 – 7.11 (m, 1H), 6.44 – 6.36 (m, 2H), 6.25 (s, 5H), 6.03 – 5.98 (m, 1H), 4.77 (dd, J = 15.1 Hz, J = 6.1 Hz, 1H), 4.68 (d, J = 13.9 Hz, 1H), 4.53 (d, J = 13.9 Hz, 1H), 4.39 (dd, J = 15.1 Hz, J = 4.8 Hz, 1H), 1.82 (s, 3H), 1.78 – 1.14 (m, 7H), 1.11 (s, 3H), 0.89 (t, J = 7.1 Hz, 3H), 0.82 (t, J = 7.1 Hz, 3H), 0.69 – 0.55 (m, 1H); ^{13}C-NMR (75 MHz, $CDCl_3$): δ = 184.4, 150.1, 135.9, 133.2, 129.8, 128.9, 128.0, 127.4, 125.7, 121.1, 120.7, 117.7, 110.7, 52.7, 48.5, 46.9, 42.8, 38.8, 38.8, 23.7, 21.9, 18.0, 17.6, 15.2, 14.9; MS (8.0 eV, ESI): m/z (%) = 541.2 (100), 537.3 (84), 523.3 (5), 445.2 (40), 431.2 (10); HRMS (8.0 eV, ESI): calcd. for $C_{30}H_{41}N_4O_2{}^{46}Ti^+$: 535.2750; found 535.2753 $C_{30}H_{41}N_4O_2{}^{46}Ti^+$ = $[M – 2Cl + OCH_3]^+$; IR: ATR, v [cm^{-1}] = 2960, 2870, 2095, 1575, 1520, 1360, 1255, 1010, 825, 725; Crystallized from CH_2Cl_2, Anal. calcd. for $C_{29}H_{38}Cl_2N_4OTi$ (CH_2Cl_2): C 59.65, H 6.57, N 13.31; found: C 59.73, H 6.37, N 13.22.

General Procedure for the Synthesis of Triazoles from Azides

To a solution of the carboxylate (1 equiv.) in CH_2Cl_2 (10 mL/mmol) was added cyclooctyne (5 equiv.). After stirring for 16 h at r.t. the solvent was removed and the residue washed with cyclohexane and chromatographed on BioBeads S-X3 to yield the desired product.

Synthesis of **12**: Azide **4** (99 mg, 0.22 mmol) with cyclooctyne (119 mg) to yield **12** (97 mg, 80%);

M.p.: 82 °C; ^{1}H-NMR (400 MHz, $CDCl_3$): *δ* = 12.07 (br. s, 1H), 7.24 – 7.17 (m, 1H), 6.82 – 6.74 (m, 1H), 6.66 (s, 5H), 6.59 – 6.52 (m, 1H), 5.93 – 5.86 (m, 1H), 4.27 – 4.19 (m, 2H), 3.35 – 3.20 (m, 2H), 3.16 (d, *J* = 14.4 Hz, 1H), 2.84 (m, 3H), 2.77 - 2.71 (m, 2H), 1.99 – 1.88 (m, 2H), 1.82 – 1.74 (m, 2H), 1.71 – 1.56 (m, 4H), 1.51 – 1.34 (m, 4H), 1.25 (s, 3H), 1.19 (s, 3H); ^{13}C-NMR (100 MHz, $CDCl_3$): *δ* = 176.5, 149.6, 144.3, 133.7, 125.6, 121.3, 119.2, 117.4, 109.0, 47.2, 45.7, 40.9, 34.4, 29.5, 28.3, 27.3, 26.9, 26.3, 26.0, 25.5, 24.8, 24.4, 21.7; MS (8.0 eV, ESI): m/z (%) = 517.2 (7), 513.3 (100), 499.3 (3), 375.2 (3); HRMS (8.0 eV, ACN, ESI): calcd. for $C_{27}H_{38}ClN_4O^{48}Ti^+$: 517.2211, found 517.2207 $[M – Cl]^+$; IR: ATR, *v* [cm^{-1}] = 2925, 2850, 1610, 1550, 1440, 1370, 1290, 1200, 825, 725; Crystallized from CH_2Cl_2, Anal. calcd. for $C_{27}H_{38}Cl_2N_4OTi$ (CH_2Cl_2): C 52.68, H 6.32, N 8.78; found: C 52.69, H 6.55, N 8.74.

Synthesis of **13**: Azide **4** (220 mg, 0.4 mmol) with cyclooctyne (216 mg) to yield **13** (231 mg, 88%);

M.p.: >200 °C; ^{1}H-NMR (400 MHz; $CDCl_3$): 12.00 (br. s, 1H), 7.15 - 7.08 (m, 1H), 7.04 - 6.92 (m, 1H), 6.75 (s, 5H), 6.65 - 6.55 (m, 1H), 6.11 - 5.94 (m, 1H), 4.71 - 4.52 (m, 2H), 4.01 - 3.95 (m, 2H), 3.77 (d, *J* = 9.8 Hz, 1H), 3.69 - 3.48 (m, 10H), 3.40 - 3.34 (m, 1H), 3.25 - 3.19 (m, 1H), 3.07 - 3.03 (m, 2H), 2.93 - 2.89 (m, 2H), 2.82 - 2.74 (m, 1H), 1.93 - 1.78 (m, 4H), 1.52 (m, 4H), 1.31 (s, 6H), ^{13}C-NMR (100 MHz; $CDCl_3$): *δ* = 176.2, 150.4, 142.6, 136.5, 124.7, 121.4, 120.4, 116.6, 109.3, 70.7, 70.7, 70.6, 70.5, 69.6, 68.6, 49.3, 46.7, 41.6, 34.4, 29.7, 27.9, 26.5, 26.0, 25.9, 24.6, 23.6, 22.0; MS (7.0 eV, ESI): m/z (%) = 617.3 (100); HRMS (8.0 eV, ESI): calcd. for $C_{32}H_{48}ClN_4O_5{}^{47}Ti^+$: 651.2793 found: 651.2791 $[M – Cl + OCH_3]^+$; IR: ATR, *v* [cm^{-1}] = 3379, 2023, 1615, 1556, 1102, 826; Crystallized from CH_2Cl_2, Anal. calcd. for $C_{31}H_{36}Cl_2N_4O_4Ti$ (CH_2Cl_2): C 51.77, H 6.52, N 7.55, found: C 51.76, H 6.52, N 7.57.

Synthesis of **14**: Azide **6** (115 mg, 0.23 mmol) with cyclooctyne (120 mg) to yield **14** (126 mg, 78%);

M.p. 152 °C; ^{1}H-NMR (400 MHz, $CDCl_3$): *δ* = 12.81 – 12.73 (m, 1H), 7.38 (d, *J* = 8.0 Hz, 2H), 7.13 (d, *J* = 8.0 Hz, 2H), 7.00 – 6.94 (m, 1H), 6.88 – 6.79 (m, 1H), 6.54 – 6.48 (m, 1H), 6.45 (s, 5H), 6.03 – 5.96 (m, 1H), 5.44 (s, 2H), 4.47 (dd, *J* = 14.6 Hz, *J* = 6.2 Hz, 1H), 4.32 (dd, *J* = 14.6 Hz, *J* = 5.3 Hz, 1H), 3.27 (d, *J* = 14.3 Hz, 1H), 2.96

– 2.87 (m, 3H), 2.63 – 2.59 (m, 2H), 1.76 - 1.68 (m, 2H), 1.60 – 1.52 (m, 2H), 1.43 – 1.32 (m, 4H), 1.25 (s, 3H), 1.19 (s, 3H); ^{13}C-NMR: (100 MHz, $CDCl_3$): *δ* = 176.6, 150.5, 145.2, 136.6, 135.2, 133.6, 128.6, 127.6, 124.8, 121.2, 120.6, 116.4, 109.4, 51.5, 46.8, 44.7, 34.8, 29.9, 28.2, 26.2, 26.1, 25.9, 24.7, 24.6, 21.9; MS (8.0 eV, ESI): m/z (%) = 561.3 (100); HRMS (8.0 eV, ESI): calcd. for $C_{32}H_{41}N_4O_2{}^{47}Ti^+$: 560.2742, found 560.2754 [M – 2Cl + OCH_3]$^+$); IR: ATR, *v* [cm^{-1}] = 2920, 2850, 1605, 1555, 1435, 1370, 1020, 825, 600, 560; Crystallized from CH_2Cl_2, Anal. calcd. for $C_{31}H_{38}Cl_2N_4OTi$ (CH_2Cl_2): C 56.00, H 5.87, N 8.16, found: C 56.02, H 5.90, N 8.10.

Synthesis of **15**: Azide **8** (267 mg, 0.5 mmol) with cyclooctyne (270 mg) to yield **15** (295 mg, 79%);

M.p.: 174 °C; ^{1}H-NMR (400 MHz, CDCl3): *δ* = 12.90 (t, *J* = 5.8 Hz, 1H), 7.39 (d, *J* = 8.0 Hz, 2H), 7.13 (d, *J* = 8.0 Hz, 2H), 6.90 (m, 1H), 6.84 (m, 1H), 6.45 (m, 1H), 6.43 (s, 5H), 6.13 (s, 1H), 5.44 – 5.41 (s, 2H), 4.45 (dd, *J* = 14.3 Hz, *J* = 6.0 Hz, 1H), 4.29 (dd, *J* = 14.4 Hz, *J* = 5.2 Hz, 1H), 3.43 (d, *J* = 13.5 Hz, 1H), 2.90 – 2.87 (m, 2H), 2.70 (d, *J* = 13.6 Hz, 1H), 2.63 – 2.57 (m, 2H), 1.77 – 1.49 (m, 10H), 1.45 – 1.26 (m, 7H), 1.17 – 1.06 (m, 1H); ^{13}C-NMR (100 MHz, CDCl3): *δ* = 176.6, 150.3, 145.1, 136.8, 135.2, 133.6, 128.7, 127.5, 125.1, 122.0, 121.1, 115.6, 110.7, 51.5, 46.6, 44.7, 38.6, 38.4, 34.1, 28.2, 26.1, 25.8, 25.3, 24.7, 24.5, 22.2, 21.9, 21.7; MS (8.0 eV, ESI): m/z (%) = 635.3 (2), 601.3 (100), 463.2 (2); HRMS (8.0 eV, ESI): calcd. for $C_{35}H_{45}N_4O_2{}^{46}Ti^+$: 599.3063; found: 599.3063 [M – 2Cl + OCH_3]+); IR: ATR, *v* [cm^{-1}] = 2925, 2850, 1605, 1555, 1435, 1375, 1235, 1020, 915, 825, 725; Crystallized from CH_2Cl_2, Anal. calcd. for $C_{34}H_{42}Cl_2N_4OTi$ (CH_2Cl_2): C 56.63, H 6.00, N 7.49, found: C 56.75, H 5.99, N 7.40.

Synthesis of **16**: Azide **9** (267 mg, 0.5 mmol) with cyclooctyne (270 mg) to yield **16** (279 mg, 79%);

M.p.: 178 °C; ^{1}H-NMR (400 MHz, $CDCl_3$): *δ* = 12.74 (br. s, 1H), 7.49 (d, *J* = 7.4 Hz, 1H), 7.36 (dd, *J* = 7.5 Hz, *J* = 7.5 Hz, 1H), 7.26 (dd, *J* = 7.5 Hz, *J* = 7.5 Hz, 1H), 7.09 – 6.99 (m, 1H), 6.91 (d, *J* = 7.5 Hz, 1H), 6.92 – 6.86 (m, 1H), 6.50 (s, 5H), 6.41 (m, 1H), 6.15 (m, 1H), 5.74 (d, *J* = 15.7 Hz, 1H), 5.61 (d, *J* = 15.7 Hz, 1H), 4.66 (dd, *J* = 14.8 Hz, *J* = 6.4 Hz, 1H), 4.32 (dd, *J* = 14.8 Hz, *J* = 4.6 Hz, 1H), 3.30 (d, *J* = 13.5 Hz, 1H), 2.93 – 2.89 (m, 2H), 2.92 – 2.83 (m, 1H), 2.78 - 2.69 (m, 1H), 2.47 (d, *J* = 13.5 Hz, 1H), 1.83 – 1.58 (m, 8H), 1.56 – 1.27 (m, 9H), 1.15 – 1.01 (m, 1H); ^{13}C-NMR (100 MHz, $CDCl_3$) *δ* = 176.8, 150.7, 144.7, 135.3, 134.8, 133.3, 130.7, 129.3, 128.8, 124.2, 123.2, 121.4, 114.8, 110.2, 50.2, 47.6, 42.9, 38.9, 38.5, 33.8, 28.4, 26.3, 25.7, 25.3, 24.7, 24.6, 22.3, 22.2, 21.8; MS: (8.0 eV, ESI): m/z (%) = 601.3 (100), 450.2 (2); HRMS (8.0 eV, ESI): calcd. for $C_{35}H_{45}N_4O_2{}^{46}Ti^+$: 599.3063; found: 599.3067 [M – 2Cl + OCH_3]$^+$; IR: ATR, *v* [cm^{-1}] = 2925, 2850, 1600, 1555, 1440, 1375, 1235, 1015, 825, 730, 685; Crystallized from CH_2Cl_2, Anal. calcd. for $C_{34}H_{42}Cl_2N_4OTi$ (CH_2Cl_2): C 59.46, H 6.24, N 7.99; found: C 59.41, H 6.43, N 7.94.

Synthesis of **17**: Azide **10** (267 mg, 0.5 mmol) with cyclooctyne (270 mg) to yield **17** (341 mg, 92%);

M.p.: >200 °C; ^{1}H-NMR (400 MHz; $CDCl_3$): δ = 11.00 (br. s, 1H), 7.35 - 7.30 (m, 1H), 6.71 (s, 5H), 6.56 - 6.50 (m, 1H), 6.44 - 6.40 (m, 1H), 5.96 - 5.86 (m, 1H), 4.43 - 4.33 (m, 2H), 3.90 - 3.77 (m, 3H), 3.73 - 3.60 (m, 3H), 3.60 - 3.53 (m, 2H), 3.53 - 3.38 (m, 6H), 2.95 - 2.87 (m, 2H), 2.82 - 2.77 (m, 2H), 1.79 (m, 2H), 1.76 - 1.69 (m, 4H), 1.66 (s, 3H), 1.62 - 1.38 (m, 8H), 1.31 - 1.18 (m, 2H), 1.12 (s, 3H), 0.86 (t, *J* = 7.1 Hz, 3H), 0.80 (t, *J* = 7.1 Hz, 3H), 0.66 - 0.56 (m, 1H), ^{13}C-NMR (400 MHz; $CDCl_3$): δ = 183.1, 149.7, 143.9, 134.9, 126.5, 120.9, 119.8, 117.6, 110.0, 70.7, 70.5, 70.3, 70.2, 69.8, 68.9, 48.1, 47.6, 46.5, 42.2, 39.4, 39.1, 28.4, 26.3, 26.0, 24.8, 24.4, 23.9, 21.8, 21.7, 17.7, 17.5, 15.2, 14.9; MS (8.0 eV, ESI): m/z (%) = 701.4 (100), 687.4 (44); HRMS (8.0 eV, ESI): calcd. for $C_{39}H_{61}N_4O_5{}^{48}Ti^+$: 701.4121, found: 701.4120 [M – 2Cl + $OCH_3]^+$; IR: ATR, *v* [cm^{-1}] = 2923, 2850, 1599, 1555, 1443, 1378, 1235, 1015; Crystallized from CH_2Cl_2, Anal. calcd. for $C_{37}H_{58}Cl_2N_4O_4Ti$ (CH_2Cl_2): C 55.22, H 7.36, N 6.78; found: C 55.20, H 7.33, N 7.76.

Synthesis of **18**: Azide **11** (289 mg, 0.5 mmol) with cyclooctyne (270 mg) to yield **18** (257 mg, 75%);

M.p.: 181 °C; ^{1}H-NMR (400 MHz, $CDCl_3$): δ = 11.95 (br. s, 1H), 7.44 (d, *J* = 7.3 Hz, 1H), 7.32 (dd, *J* = 7.2 Hz, *J* = 7.2 Hz, 1H), 7.22 (dd, *J* = 7.2 Hz, *J* = 7.2 Hz, 1H), 7.11 – 7.06 (m, 1H), 6.72 (d, *J* = 7.5 Hz, 1H), 6.43 – 6.40 (m, 2H), 6.39 (s, 5H), 6.06 – 6.02 (m, 1H), 5.89 (d, *J* = 16.2 Hz, 1H), 5.55 (d, *J* = 16.0 Hz, 1H), 4.93 (dd, *J* = 14.7 Hz, *J* = 6.4 Hz, 1H), 4.44 (dd, *J* = 14.8 Hz, *J* = 4.0 Hz, 1H), 2.98 – 2.87 (m, 2H), 2.84 – 2.70 (m, 2H), 1.79 (s, 3H), 1.76 – 1.41 (m, 11H), 1.33 – 1.13 (m, 4H), 1.10 (s, 3H), 0.86 (t, *J* = 7.1 Hz, 3H), 0.83 (t, *J* = 7.1 Hz, 3H), 0.62 (m, 1H); ^{13}C-NMR (100 MHz, $CDCl_3$): δ = 184.4, 150.3, 145.1, 134.4, 134.3, 133.7, 128.6, 128.5, 128.5, 127.8, 125.7, 121.2, 120.9, 117.5, 110.5, 49.2, 48.6, 47.0, 43.2, 38.9, 38.8, 28.4, 26.2, 26.1, 24.8, 24.7, 23.8, 21.9, 21.9, 18.2, 17.7, 15.1, 15.0; MS (8.0 eV, ESI): m/z (%) = 645.4 (100), 631.3 (3); HRMS (8.0 eV, ESI): calcd. for $C_{38}H_{53}N_4O_2{}^{48}Ti^+$: 645.3646; found: 645.3646 [M – 2Cl + $OCH_3]^+$; IR: ATR, *v* [cm^{-1}] = 2930, 2870, 1570, 1520, 1440, 1400, 1360, 1235, 1195, 1050, 1010, 825, 730; Crystallized from CH_2Cl_2, Anal. calcd for $C_{37}H_{50}Cl_2N_4OTi$ (CH_2Cl_2): C 59.23, H 6.80, N 7.27; found: C 59.16, H 6.88, N 7.28.

Figure S1: Apoptosis-induction by **15**

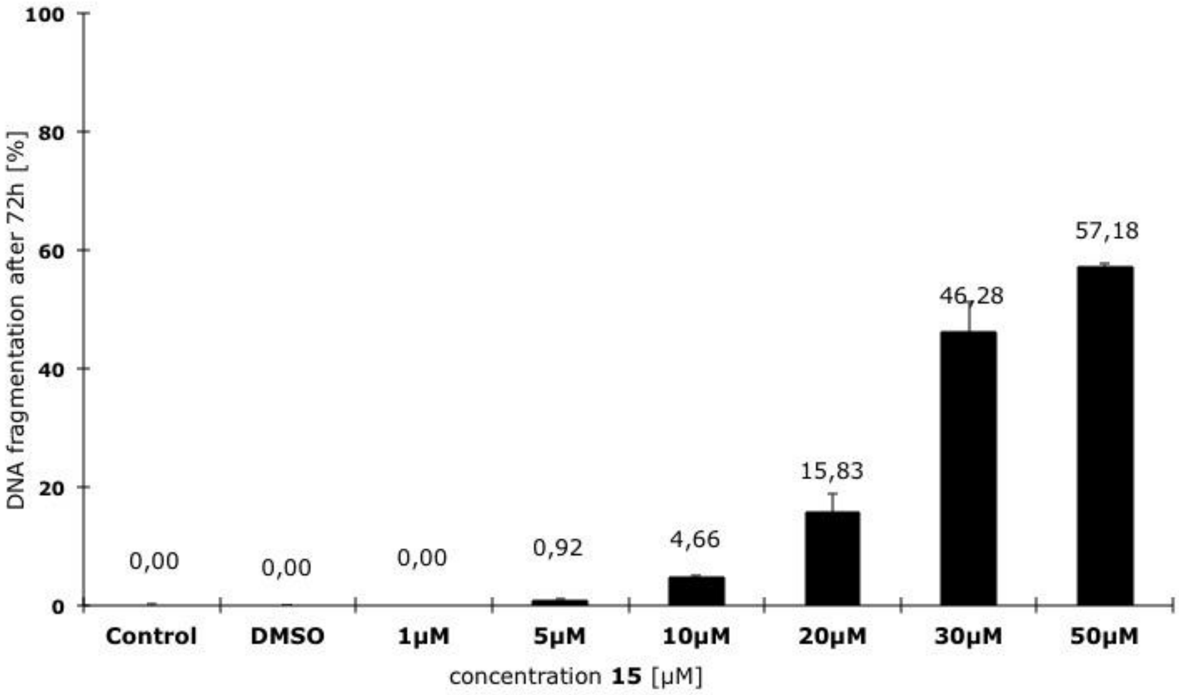

Figure S2: Viability of **15**

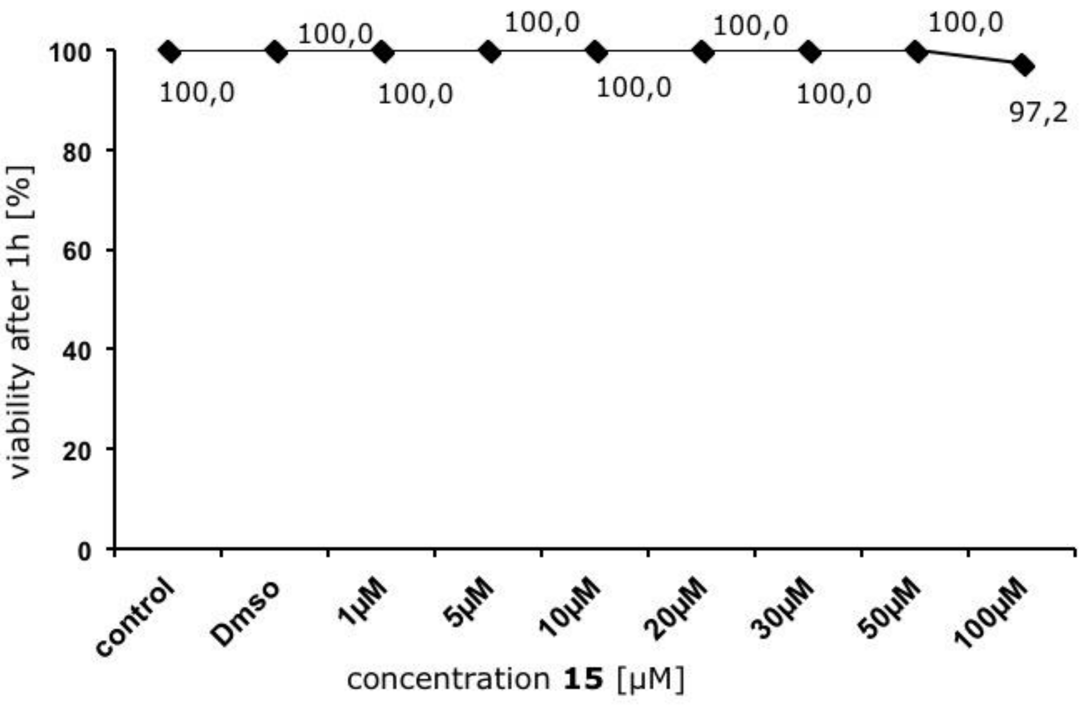

Figure S3: Apoptosis-induction by **16**

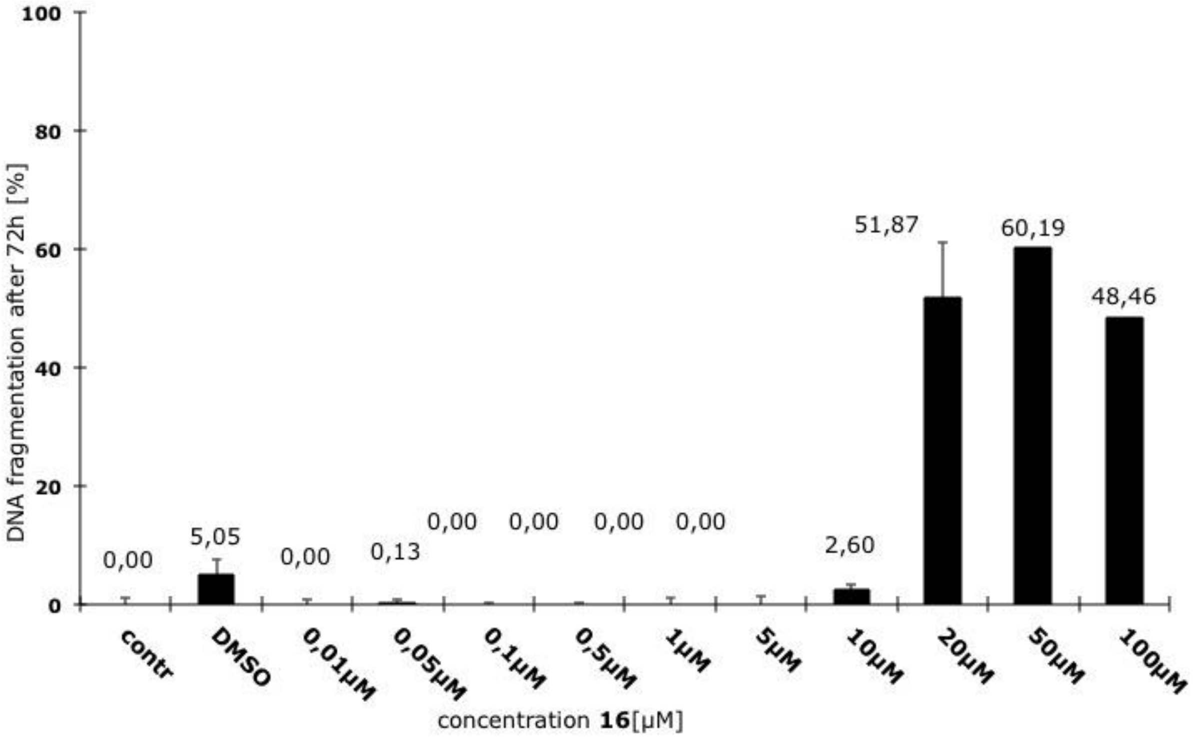

Figure S4: Viability of **16**

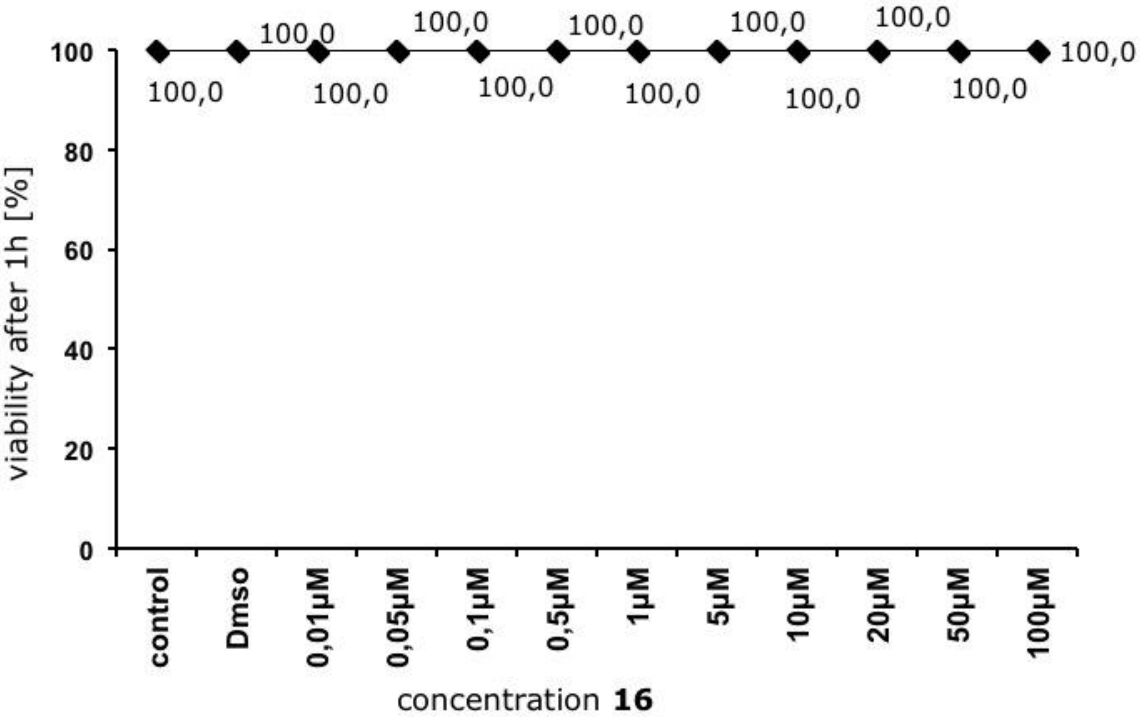

Figure S5: Apoptosis-induction by **18**

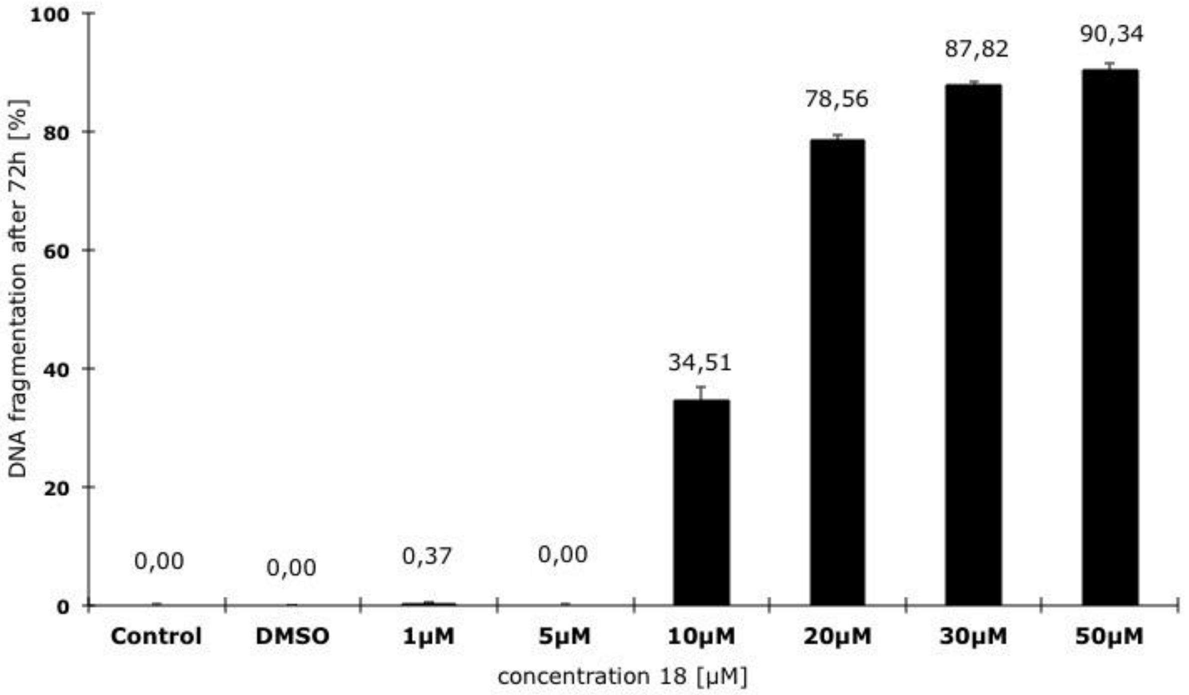

Figure S6: Viability of **18**

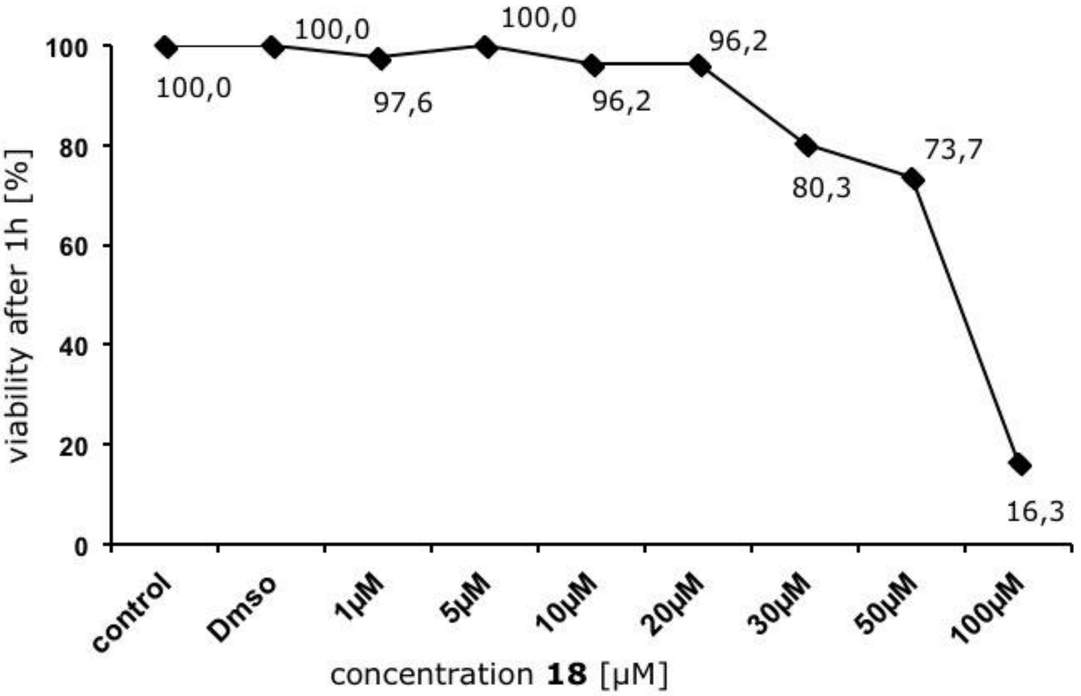

References:

[1] Thomas, J. R.; Liu, X.; Hergenrother, P. J. *J. Am. Chem. Soc.* **2005**, *127*, 12434–12435.

[2] Hou, Z.-S.; Tan, Y.-B.; Kim, K.; Zhou, Q.-F. *Polymer* **2006**, *47*, 742–750.

[3] Lau, K.-N.; Chow, H.-F.; Chan, M.-C.; Wong, K.-W. *Angew. Chem. Int. Ed.* **2008**, *47*, 6912–6916.

[4] Risseeuw, M. D. P.; De Clercq, D. J. H.; Lievens, S.; Hillaert, U.; Sinnaeve, D.; Van den Broeck, F.; Martins, J. C.; Tavernier, J.; Van Calenbergh, S. *ChemMedChem* **2013**, *8*, 521–526.

[5] Shults, E. E.; Velder, J.; Schmalz, H.-G.; Chernov, S. V.; Rubalova, T. V.; Gatilov, Y. V.; Henze, G.; Tolstikov, G. A.; Prokop, A. *Bioorg. Med. Chem. Lett.* **2006**, *16*, 4228-4232.

[6] Essmann, F.; Wieder, T.; Otto, A.; Müller, E.-C.; Dörken, B.; Daniel, P. T. *Biochem. J.* **2000**, *346*, 777-783.

[7] Yang, J.; Li, H.; Chen, Y.-Y; Wang, X.-J.; Shi, G.-Y.; Hu, Q.-S.; Kang, X.-L.; Lu, Y.; Tang, X.-M.; Guo, Q.-S.; Yi, J. *Free Radical Biol. Med*. **2004**, *37*, 2027-2041.
